# Experimental Study of Thermal and Fire Reaction Properties of Glass Fiber/Bismaleimide Composites for Aeronautic Application

**DOI:** 10.3390/polym15102275

**Published:** 2023-05-11

**Authors:** Gang Li, Fang Qu, Zhi Wang, Xuhai Xiong, Yanying Xu

**Affiliations:** 1 Fire & Explosion Protection Laboratory, Northeastern University, Shenyang 110819, China; ligang@mail.neu.edu.cn; 2 Liaoning Key Laboratory of Aircraft Fire Explosion Control and Reliability Airworthiness Technology, Shenyang Aerospace University, Shenyang 110136, China; 3 Liaoning Key Laboratory of Advanced Polymeric Composites, Shenyang Aerospace University, Shenyang 110136, China; xiongxuhai@126.com

**Keywords:** glass fiber (GF)/bismaleimide (BMI) composites, pyrolysis, heat release, limiting oxygen index, specific optical density

## Abstract

Thermal behavior and fire reaction properties of aerial glass fiber (GF)/bismaleimide (BMI) composites were tested using thermogravimetric analysis (TGA), thermogravimetric coupled with Fourier transform infrared spectroscopy (TG-FTIR), cone calorimeter, limiting oxygen index, and smoke density chamber. The results showed that the pyrolysis process was one stage in a nitrogen atmosphere with the prominent volatile components of CO_2_, H_2_O, CH_4_, NO_x_, and SO_2_. The release of heat and smoke increased with the increase in heat flux, while the time required to reach hazardous conditions decreased. The limiting oxygen index decreased monotonically from 47.8% to 39.0% with increasing experimental temperature. The maximum specific optical density within 20 min in the non-flaming mode was greater than that in the flaming mode. According to the four kinds of fire hazard assessment indicators, the greater the heat flux, the higher the fire hazard, for the contribution of more decomposed components. The calculations of two indices confirmed that the smoke release in the early stage of fire was more negative under flaming mode. This work can provide a comprehensive understanding of the thermal and fire characteristics of GF/BMI composites used for aircraft.

## 1. Introduction

Fiber-reinforced resin matrix composites have remarkable advantages, such as light weight, high strength, flexible designability, fatigue, and corrosion resistance, and easy monolithic molding, and have been increasingly applied in aerospace, weapons, electric, marine, and automotive industries [1,2,3,4,5,6,7,8,9,10]. Among them, glass fiber (GF) is the first low-cost reinforcement adopted for the preparation of resin matrix composites for aircraft [11,12] due to its heat and fire resistance, high tensile strength, good electrical insulation, and chemical stability. Its commercialization is now relatively mature. On the other hand, there are three main types of high-performance matrix resins currently used as fiber-reinforced aerial composites: epoxy resin, bismaleimide (BMI) resin, and polyimide resin. Epoxy resin has excellent processability but poor heat and moisture resistance and has gradually failed to meet the requirements of the growth of advanced composites. Polyimide resin has outstanding thermal stability as well as heat and moisture resistance, but its high cost and harsh molding process conditions limit its wide application. However, BMI resin not only has many excellent properties similar to polyimide resin, such as high temperature resistance, radiation resistance, heat, and moisture resistance, but also has excellent processing properties similar to epoxy resin after modification, which meets the development requirements of aerial advanced composites in many aspects, especially in primary and secondary load-bearing structures. Therefore, BMI resin plays an increasingly prominent role in matrix applications for aircraft composites. At present, the main matrix resins in GF composites are epoxy and BMI. GF/epoxy composites are usually applied for control surfaces, fairings, radomes, and rotor blades of aircraft [13], comparatively, GF/BMI composites are mainly used for interior decorations, high performance radomes, electrochemical corrosion resistant liners, and auxiliary power unit panels [14,15,16]. However, compared to traditional metal materials used in aircraft, although GF has excellent fireproof and fire-resistant performance, BMI can decompose, even burn, and release toxic fumes when exposed to high temperature or flame, even though no melt drops are produced. These hazards not only increase the fire load and easily cause poisoning or asphyxiation but also lead to a reduction in structural strength, seriously compromising the safety of the aircraft and its occupants. Therefore, more attention should be paid to the fire safety of aircraft caused by GF/BMI composites.

Most studies have focused on changes in the mechanical properties of GF/BMI composites [17,18,19,20,21,22]. There has also been some progress on the effect of the flame retardant modification applied to the copper-clad matrix on the flammability and mechanical and dielectric properties of GF/BMI composites [8,23,24]. In contrast, research on the thermal behavior and fire reaction properties of GF/BMI composites for aeronautic applications is fairly limited [13,25]. Drukker et al. [26] investigated the chemical, physical, and mechanical properties of GF/BMI laminates with varying laminate thicknesses exposed to high temperatures, and found that the interlaminar shear strength decreased, the glass transition temperature increased, the resin underwent chemical changes, and the degradation also became more significant at higher temperatures as well. Razgon and Sukenik [27] utilized TGA to analyze how titanium dioxide films coated on the surface of GF/BMI composites could prevent the pyrolysis of the composite when exposed to an oxidizing atmosphere at high temperatures, thus reducing the damage to the integrity of the composites. Kourtides et al. [28] tested the thermomechanical properties and flammability of some fiber-reinforced thermoplastic and thermoset laminates for aircraft interiors, and 181 glass cloth/BMI composites were found to have the highest ambient-temperature oxygen index and low flaming specific optical density, confirming the good fire safety of GF/BMI composites. Kourtides et al. [29] also tested the flammability of some sandwich structure panels for aircraft interiors, where the combined structure of 181 glass cloth/BMI composites and 182 glass cloth/BMI composites, as well as honeycomb filled with quinone dioxime foam, had a slightly higher ambient-temperature oxygen index, a higher flaming specific optical density than that of 181 cloth/BMI composites, and a relatively higher total heat release at both heat fluxes among four kinds of composites. In the end, the GF/BMI sandwich panels were considered to exhibit higher fire containment ability owing to the foam in the core.

However, with the toughened modification of BMI and the development in the processing technology of GF/BMI composites, more detailed and comprehensive work is needed to determine the specific performance of different GF compositions and the ratios of GF/BMI composites when exposed to high temperatures and fires. Therefore, in this paper, simultaneous thermal analyzer, thermogravimetric coupled with Fourier transform infrared spectroscopy (TG-FTIR) test device, cone calorimeter, limiting oxygen index tester, and smoke density chamber were used to study the thermal behavior and combustion characteristics of GF/BMI composites for aircraft under different fire conditions. Furthermore, four kinds of fire hazard assessment indicators based on cone calorimeter tests and two indices drawn from smoke density tests were compared and analyzed. The experiments can provide data support for the perfection of relevant standards and provisions for fire protection section in airworthiness, and useful guidance for the fire hazard assessment in subsequent research and application of new GF/BMI composites in different aeronautic parts.

## 2. Materials and Methods

### 2.1. Materials

**Figure 1:**
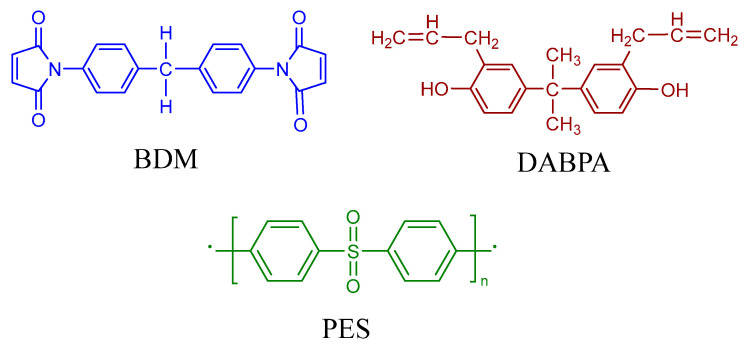
Chemical structures of BDM, DABPA, and PES.

GF/BMI composites selected in the study were provided by Youpute Technology Co., Ltd., Shenyang, China. The matrix is a kind of commercial grade resin with the main composition of N,N′-4,4′-bismaleimdodiphenylmethane (BDM) and 2,2-diallylbisphenol A (DABPA), as well as polyether sulfone (PES). Figure 1 shows the chemical structures of BDM, DABPA, and PES. The laminate was manufactured by 20 plies of woven GF reinforced BMI pregregs at an orientation and cured in an autoclave (Xi’an Chemical General Machine Factory, Xi’an, China) with a vacuum bag. Then, the fabricated laminate with a fiber volume of 60% was cut into the required specimen specifications through mechanical processing.

### 2.2. Characterization and Measurement

Thermogravimetric analysis (TGA) was accomplished with a SHIMADZU DTG-60(AH) simultaneous thermal analyzer (SHIMADZU, Kyoto, Japan) in a highly purified nitrogen atmosphere. The flow rate of the carrier gas was set to 50 mL/min. The dried specimen (approximately 5 mg in block shape) chosen for the experiment was put into an alumina crucible and heated from 40 to 1000 °C at five heating rates of 5, 10, 20, 30, and 40 °C/min for each test. The gases evolved during pyrolysis at a heating rate of 10 °C/min in a nitrogen atmosphere were traced using TG-FTIR test device consisting of a NETZSCH STA-2500 TG analyzer (NETZSCH, Selb, Germany) and a Thermo Fisher Nicolet iS50 FTIR spectrometer (Thermo Fisher Scientific, Massachusetts, USA). The spectra were recorded with a spectral resolution of 4 cm^−1^ and an average of 32 scans over the 4000–450 cm^−1^ wavenumber range. Cone calorimeter tests were performed using a FTT-CONE-0242 cone calorimeter (Fire Testing Technology, East Grinstead, UK) according to ISO 5660-1. The heat fluxes were set as 25, 35, and 50 kW/m^2^ to represent different fire scenarios. The size of the specimen was 100 mm × 100 mm × 2 mm, and the outer edge of which was wrapped with aluminum foil before the test. Limiting oxygen index (LOI) tests were conducted using an FTT-OL-1402072 LOI apparatus (Fire Testing Technology, East Grinstead, UK) in accordance with ISO 4589-2 and ISO 4589-3. The specimens with dimensions of 150 mm × 10 mm × 2 mm were tested with temperatures varying from ambient temperature to elevated temperature up to 220 °C. A FTT-SDC-1411510 smoke density chamber (Fire Testing Technology, East Grinstead, UK) was used to measure specific optical density and light transmittance according to ASTM E662 in flaming and non-flaming conditions [30]. The flat specimen was exposed to a radiant heat source of 25 kW/m^2^ with or without the use of a pilot flame, and the raw specimen had a size of 75 mm × 75 mm × 2 mm with outer edge wrapped with aluminum foil before testing.

## 3. Results

### 3.1. Thermal Decomposition Characteristics

**Figure 2:**
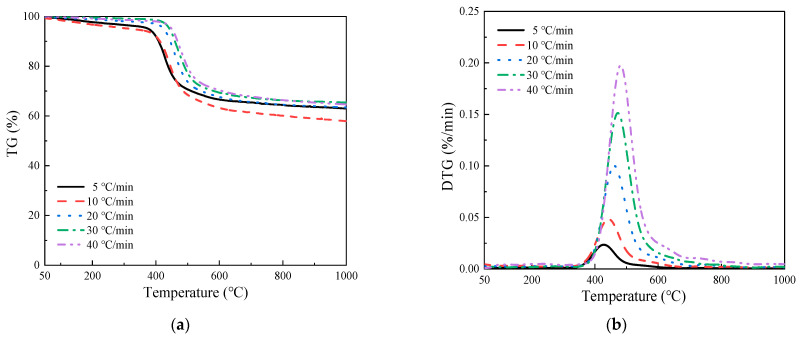
(**a**) TG and (**b**) DTG curves of GF/BMI composites at various heating rates.

The premise of solid combustion is pyrolysis, and the study of pyrolysis characteristics is the basis for the study of solid combustion characteristics and fire propagation [31]. Figure 2 shows the thermogravimetric (TG) and derivative thermogravimetric (DTG) curves of GF/BMI composites at various heating rates under a nitrogen atmosphere. The TG and DTG curves basically followed the same pattern with the increase of heating rate. It can be clearly seen that only one distinct inflection point appeared in all TG curves, which corresponded to the respective peak of the DTG curve. Therefore, a one-stage process was manifested in the thermal decomposition of GF/BMI composites because GF could not react within the pyrolysis temperature interval in the inert atmosphere [25], only the BMI in GF/BMI composites underwent thermal decomposition. At the same time, the residual weight around 60% in the TG curves also confirmed this phenomenon. In general, the thermal decomposition of the polymers caused by high-temperature cracking was dominated by random chain breakage, which occurred first in the weak part of the resin molecular chain [32], whereas the imide ring was basically the thermally weak link in the backbone. Thus, the carbon-carbon bond of the maleimide ring was the first to decompose under thermal action, releasing CO_2_, H_2_O, and other degradation products [33,34,35].

The pyrolysis parameters, including the initial decomposition temperature T_o_, the final decomposition temperature T_f_, the maximum mass loss rate temperature T_p_, and the maximum mass loss rate MLR_p_ at different heating rate β are shown in Table 1. It can be seen that with the increase of the heating rate, the initial decomposition temperature, the final decomposition temperature, and the maximum mass loss rate temperature in the pyrolysis stage shifted to the high temperature direction, and the maximum mass loss rate also gradually increased correspondingly. In addition, the initial decomposition temperature of GF/BMI composites was significantly higher than that of GF/epoxy composites [13,25], mainly due to the high cross-linking density together with the presence of benzene and imide heterocycles in the BMI to reflect the excellent heat resistance.

### 3.2. Pyrolysis Kinetic Analysis

Although the activation energy obtained in the pyrolysis of some polymers plays a limited role [36], some researchers still use the activation energy derived from non-isothermal thermogravimetric test data to measure the difficulty of polymer pyrolysis, i.e., the activation energy is considered to be the minimum energy threshold for a certain reaction to occur, and the higher the activation energy, the more difficult the reaction is to occur [37,38,39]. In addition, more pyrolysis kinetic parameters extracted from the tests can also provide relevant references for subsequent studies. Therefore, in this study, the activation energy of GF/BMI composites during pyrolysis was calculated using model-free methods.

Model-free methods can provide relatively reliable activation energy without involving the kinetic function, avoiding errors caused by different reaction mechanism functions and becoming widely used for determining pyrolysis kinetic parameters [40]. Among many model-free methods, Kissinger–Akahira–Sunose (KAS) is a common one developed on the basis of the Kissinger method [41,42], and the equation is expressed as follows:

(1) $$\ln\left(\frac{\beta }{T^{2}}\right)=\ln\left(\frac{A\times R}{E\times g\left(\alpha \right)}\right)-\frac{E}{R\times T}$$

where *β* and *α* are the heating rate (°C/min) and the conversion rate (%), respectively. *T*, *A*, and *E* denote the absolute reaction temperature (K), the pre-exponential factor (1/s), and the activation energy (kJ/mol), respectively. R is the universal gas constant (8.314 J/mol K) and *g*(*α*) serves as the integral form of the reaction mechanism function.

**Figure 3:**
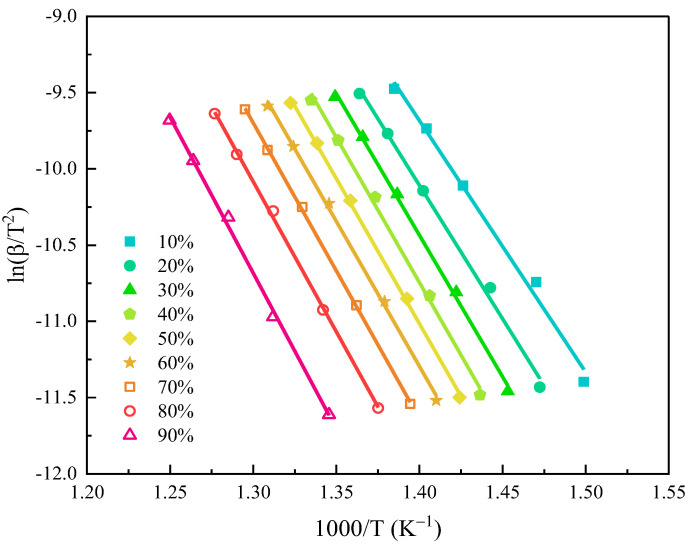
The KAS method plots for various conversion rates.

Figure 3 shows the fitting curves of ln(*β*/T^2^) against 1000/T from 10% to 90% conversion rate with a 10% increment. The fitted straight lines were integrated and substituted into Equation (1) to obtain the activation energy corresponding to each conversion rate, and the results are listed in Table 2. All fitting curves displayed high correlation coefficients R^2^ (>0.98), indicating that the activation energy calculated based on the KAS method was quite accurate. The average activation energy was up to 156 kJ/mol, which was higher than that of GF/epoxy composites [13], again demonstrating the good thermal stability of GF/BMI composites.

### 3.3. TG–FTIR Analysis

**Figure 4:**
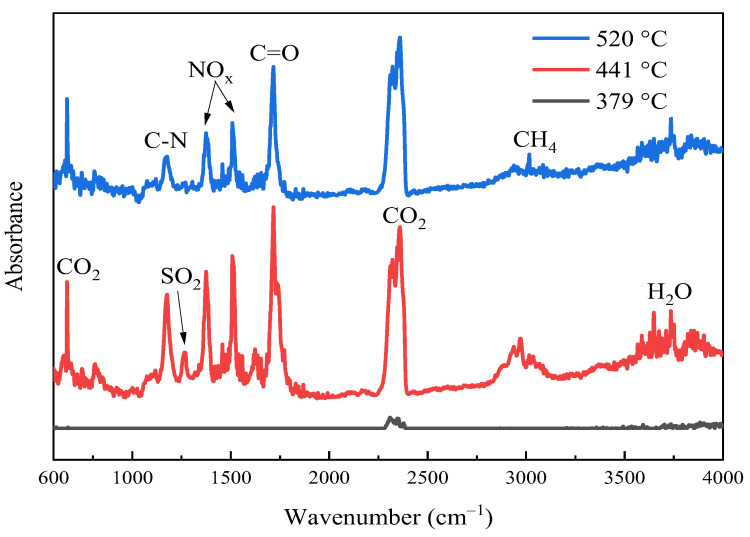
FTIR spectra of decomposition gas products from GF/BMI composites at three different temperatures evolved during TGA.

The FTIR spectra of thermal decomposition gases at T_o_, T_p_, and T_f_ in a nitrogen atmosphere with a heating rate of 10 °C/min are shown in Figure 4. At the maximum mass loss rate temperature of 441 °C, the BMI matrix decomposed most adequately. CO_2_ (670, 2300–2400 cm^−1^) was apparently detected mainly due to the cleavage of carbonyl groups of the maleimide ring, and H_2_O (3500–4000 cm^−1^) was the by-product of etherification condensation of DABPA phenolic hydroxyl groups. CH_4_ (2930–2980 cm^−1^) was a decomposed product of DABPA aliphatic chains [43]. C=O (1710 cm^−1^), NO_x_ (1373, 1509 cm^−1^), and C-N (1179 cm^−1^) were probably the fragments of the maleimide rings of BDM [44], while SO_2_ (1264 cm^−1^) was the fragment of the broken PES chains [45,46]. With a further increase in temperature, at the final decomposition temperature of 520 °C, the release of SO_2_, NO_x_, and CH_4_ decreased significantly compared to that at 441 °C.

### 3.4. Cone Calorimeter Tests

Three kinds of heat fluxes were used to simulate different fire severities. According to the principle of oxygen consumption, the burning characteristics of GF/BMI composites were investigated, and the time-to-ignition (TTI), peak heat release rate (pHRR), time-to-pHRR, total heat release (THR), total smoke release (TSR), and residual weight (RW) were chosen as the representative parameters and listed in Table 3. The TTI can be an approximate measure of the flammability of a material, i.e., the longer the ignition time, the worse the flammability, and the lower the fire hazard [47]. It is clear from Table 3 that the TTI decreased significantly as the heat flux increased. At 25 kW/m^2^, the TTI was as long as 126 s, whereas when the heat flux doubled to 50 kW/m^2^, the TTI was shortened to only 38 s, a reduction of almost 70%. This is mainly due to the increase in the rate of thermal decomposition as a result of increasing the heat flux, which leads to an increase in the heat exposure area and a rapid increase in the rate of flammable gas generation, making the material more likely to be ignited [48,49]. On the other hand, the increase in the thermal decomposition rate also led to the increase in the THR and the TSR, as well as a decrease in the RW.

**Figure 5:**
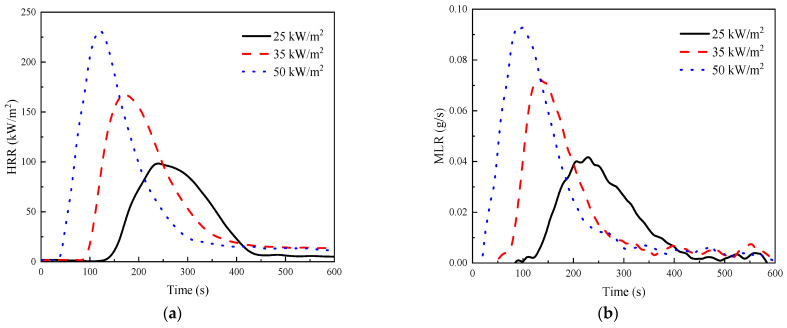
(**a**) HRR and (**b**) MLR curves of GF/BMI composites under different heat fluxes.

Figure 5 shows the heat release rate (HRR) and mass loss rate (MLR) curves of GF/BMI composites under different heat fluxes. HRR is one of the most important parameters in fire reaction characteristics and plays a crucial role in fire growth and spread [47,50,51]. With the increase in the heat flux, the overall HRR of GF/BMI composites increased in a similar pattern of variation, with a trend of rising to a peak before decreasing. The pHRR also increased with the heat flux level, while the time-to-pHRR decreased as the heat flux increased. As can be seen from Table 3, the value of pHRR was 242.0 kW/m^2^ at 116 s under 50 kW/m^2^, while it took 225 s to reach a pHRR of 104.9 kW/m^2^ under 25 kW/m^2^. These changes coincided with the increase in flammable gases caused by the advancement of ignition time at a higher heat flux level. The volatile gases were ignited and released heat after reaching a certain concentration, reaching the heat release peak when more heat accumulated, and then the heat release gradually decreased until the end due to the reduction of the consumption of combustible materials. MLR is regarded as representing real-time thermal decomposition changes during the combustion process [31]. It can be seen that the shapes of the MLR curves can all be divided into three stages. The mass loss of GF/BMI composites was slow at stage I during 38–123 s under 25 kW/m^2^. At this time, although GF/BMI composites had not been ignited yet, a trace of smoke release had begun to appear, probably because the BMI in the surface layer began to break at some weak parts of the molecular chain and reorganize into new small molecules or ionic structures, such as CO_2_, CH_4,_ and other gases [52]. At stage II, from 124 to 229 s, the MLR basically increased in a straight line, which was more consistent with the change law of the HRR. At stage III, 230 s later, the MLR started to gradually decrease and eventually stabilized. It should be the degradation of the BMI to form a char layer, which not only isolated the infiltration of air, but also prevented the diffusion of combustible gases into the combustion zone, thus inhibiting further decomposition and combustion.

**Figure 6:**
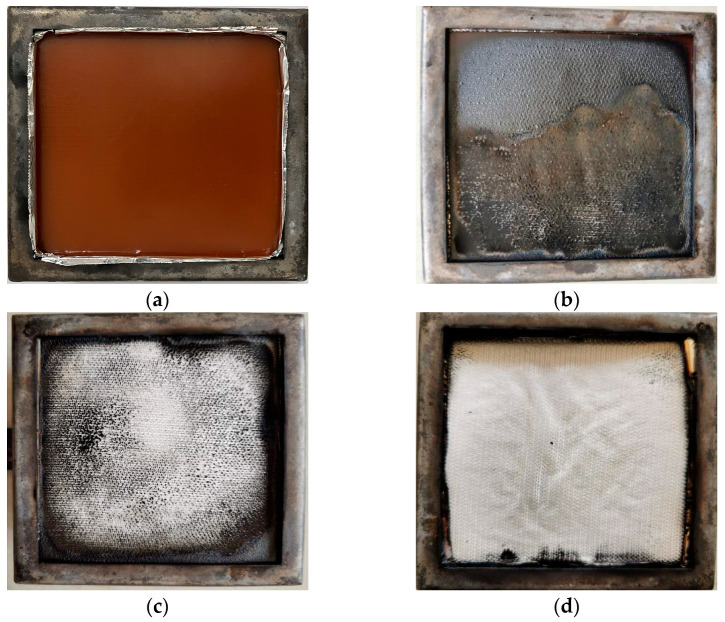
The morphologies of GF/BMI composites before and after combustion under different heat fluxes: (**a**) before combustion; (**b**) 25 kW/m^2^; (**c**) 35 kW/m^2^; (**d**) 50 kW/m^2^.

Macroscopic morphological analysis is a description of the basic situation of changes in geometry, size, and surface condition of an object. Figure 6 shows the morphologies of GF/BMI composites before and after combustion under different heat fluxes. The comparison showed that with the increase of heat flux, the pyrolysis and combustion of GF/BMI composites became more complete, and the surface charring gradually decreased until it nearly disappeared, almost the entire exposed surface was visible as GF fabric under 50 kW/m^2^. The GF fabric did not change obviously in all tests, and the shape remained basically intact without cracks. This also coincided with the RW changes mentioned above. During the combustion process, GF/BMI composites never appeared in the molten drop phenomenon. 

The fire performance index (FPI), total heat release index (THRI_6min_), total smoke production index (TSPI_6min_), and toxic gas production index (T_ox_PI_6min_) [53] were introduced to comprehensively evaluate the fire hazard characteristics of GF/BMI composites. FPI is an important indicator to characterize the potential fire hazard, the smaller the FPI, the shorter the fire initiation time, the greater the fire intensity, and the higher the possibility of flashover. THRI_6min_ is usually defined as the logarithmic value of the total amount of heat released within the first 6 min of the test, and the larger the THRI_6min_, the more heat the material releases within the specified time, the faster the fire temperature rises, and the greater the thermal damage caused. A cone calorimeter can use the laser system measuring the smoke coefficient to calculate the extinction area, so the TSPI_6min_ is usually the logarithmic value of the total smoke production within the first 6 min of the test, the greater the TSPI_6min_, the more smoke produced by the material within the specified time and the greater the smoke hazard. CO is the main toxic gas causing casualties in fires, T_ox_PI_6min_ is approximated by the product of CO yield (COY) and MLR, which is the logarithm of the CO production rate within the first 6 min of the test. Obviously, the greater the T_ox_PI_6min_, the greater the toxic smoke volume of the material within the specified time, and the higher the poisoning probability. The equations are expressed as follows:

(2) FPI=TTI/pHRR 

(3) $$THRI_{6min}=\log(HRR\times 0.36)$$

(4) $$TSPI_{6min}=\log(SEA\times MLR\times 36)$$

(5) $$T_{ox}PI_{6min}=\log(COY\times MLR\times 10^{3})$$

where *SEA* denotes the specific extinction area (m^2^/kg).

The calculated values are shown in Table 4. Comparing the above four indices under different heat fluxes, it can be seen that, except for FPI, which decreased with the increase of heat flux, the other three indices all increased gradually. This was consistent with the situation expressed by the HRR and the combustion performance parameters in Table 3, i.e., the overall fire reaction characteristics of GF/BMI composites were enhanced, and the fire hazard was increased with the increase of heat flux. Therefore, in practical applications, fireproofing, and insulation plans for different parts and zones should be designed accordingly.

### 3.5. LOI Tests

LOI is the minimum oxygen concentration required to maintain the combustion of a material when a mixture of oxygen and nitrogen is introduced, expressed as a percentage by volume. It is often used to quantify and characterize the combustibility of a material, and a higher LOI indicates that the material is less likely to burn. In order to fully reflect the influence of a fire scenario on the combustion performance of GF/BMI composites, LOI tests at ambient temperature and elevated temperature were carried out, and the results are shown in Table 5. The comparison showed that LOI always decreased slightly with increasing temperature, and the highest LOI was 47.8% at ambient temperature, which was mainly due to the intrinsic flame retardancy of the aromatic polymer structure in the BMI. However, when the experimental temperature was increased by 200 °C, the LOI decreased 81.6% to 39.0%, due to the reduction of the heat required to maintain decomposition and combustion [47]. Nevertheless, LOI at 220 °C still reached the B1 fire resistance level according to GB 8624-2012 [54], once again demonstrating the good flame retardant properties of GF/BMI composites.

### 3.6. Smoke Density Tests

**Figure 7:**
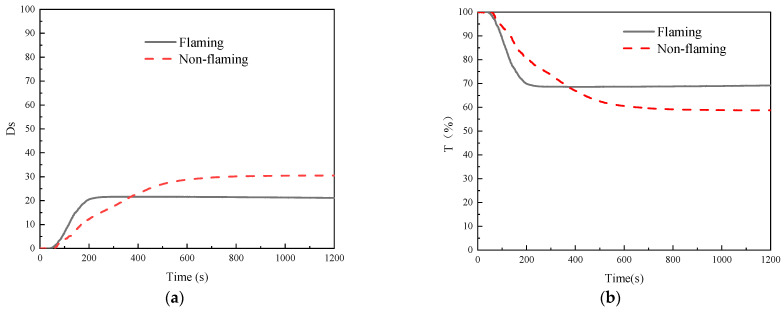
(**a**) D_S_ and (**b**) T curves of GF/BMI composites in different fire patterns.

**Figure 8:**
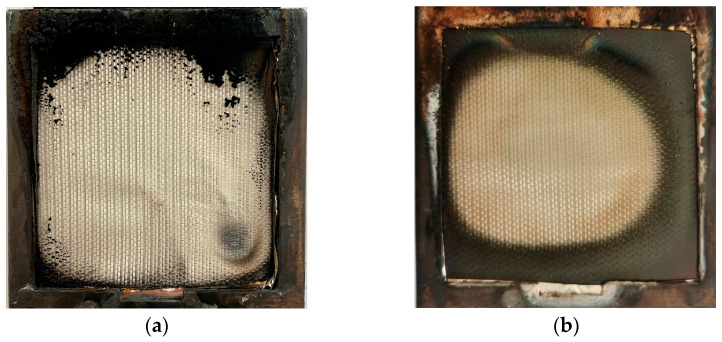
The post-test morphologies of GF/BMI composites in different fire patterns: (**a**) flaming; (**b**) non-flaming.

Smoke is an important product in the combustion process, almost all fires produce a large amount of smoke, and high temperature smoke will not only accelerate the spread of fire but also reduce the visibility of fire scenes, seriously interfering with evacuation and rescue. In a certain volume of confined space, specific optical density (D_S_) can be used to characterize the smoke-generating capacity of a material by measuring the degree of light attenuation after the light beam passes through the smoke layer generated by combustion. Smoke density tests were conducted on GF/BMI composites in flaming mode and non-flaming mode, respectively, and the changes in D_S_ and light transmittance (T) were plotted in Figure 7. The post-test morphologies of GF/BMI composites are shown in Figure 8. The smoke characteristic parameters, such as maximum specific optical density (D_m_), time to D_m_ (tD_m_), maximum specific optical density in 4 min (^4^D_m_), and time to critical specific optical density (t_16_), which is the time from the start of the test to reach a specific optical density at the light transmittance of 75%, are all listed in Table 6. At the same time, Table 6 shows two indices for smoke hazard evaluation calculated by Equations (6) and (7), one is the average accumulation rate (R), defined as the average linear rate of four 20% smoke intervals between 10% and 90% of D_m_ [55]. The other is the smoke obscuration index (SOI), which incorporates the effects of total smoke volume and smoke generation rate when the critical specific optical density is reached. In general, the higher the R or SOI, the greater the smoke hazard.

(6) $$R=\frac{D_{m}}{20}\left(\frac{1}{t_{0.3}-t_{0.1}}+\frac{1}{t_{0.5}-t_{0.3}}+\frac{1}{t_{0.7}-t_{0.5}}+\frac{1}{t_{0.9}-t_{0.7}}\right)$$

(7) $$SOI=\frac{D_{m}^{2}}{2000\;t_{16}}\left(\frac{1}{t_{0.3}-t_{0.1}}+\frac{1}{t_{0.5}-t_{0.3}}+\frac{1}{t_{0.7}-t_{0.5}}+\frac{1}{t_{0.9}-t_{0.7}}\right)$$

where *t*_0.1_, *t*_0.3_, indicates the time in min at which the smoke accumulation reaches 10%, 30%, etc., respectively, of *D_m_*.

As shown in Table 6, the D_m_ reached 30.56 in the non-flaming mode, 8.92 higher than that in the flaming mode of 21.64. This was mainly due to the fact that, in flaming mode, the overall combustion of GF/BMI composites was more adequate under the dual action of thermal radiation and flame, so the smoke concentration became lower, which was also reflected in the post-test morphologies shown in Figure 8. It can be seen from Figure 7a that GF/BMI composites started to release smoke at 43 s in flaming mode, 11 s earlier than that in non-flaming mode, and the D_S_ rose linearly rapidly from 43 to 262 s, which was mainly attributed to the fact that after contact with the flame, the thermal decomposition layer on the surface deepened into GF/BMI composites due to the continuous heat transfer, providing sufficient fuel for continuous combustion, and while releasing CO_2_, NO_x_ and other gases. Subsequently, the increase of D_S_ slowed down during 263–457 s and reached D_m_ of 21.64 at 457 s. 458 s later, the D_S_ basically kept steady with the slight reduction of matrix resin involved in combustion, and the change trend remained consistent with the results of the cone calorimeter test. The T is the percentage ratio of the projected luminous flux to the incident luminous flux, and its variation rule is exactly opposite to that of the D_S_. Therefore, as shown in Figure 7b, the variation of T in flaming mode could also be divided into three stages: the first steady stage, when GF/BMI composites did not start emitting smoke with the T of basically 100%; the second stage, when the D_S_ started to increase and the T started to decrease; and the third stage, when the D_S_ slowly decreased and the T slightly increased at the end of the test. The same was true for the D_S_ and the T variation patterns in the non-flaming mode. Figure 7 also reveals that the D_m_ and the T were both superior to the corresponding value of GF/epoxy composites in each fire pattern [13], demonstrating the excellent smoke suppression properties of GF/BMI. However, although the D_m_ was greater in the non-flaming mode, it took more time to reach this value. Similarly, the t16 as a simple measurement of the initial smoke generation was also longer than that in flaming mode, while the ^4^D_m_ was larger in flaming mode, R and SOI were also higher than those in non-flaming mode. These indicated that the smoke generation in the early stages of flaming mode was greater and had a greater impact on visibility, which was consistent with the earlier reduction of T in flaming mode. Therefore, fire conditions involving GF/BMI composites under the dual action of thermal radiation and flame should be avoided to prevent greater obstruction of evacuation and rescue of personnel.

## 4. Conclusions

In this work, an investigation of the thermal and fire behavior of GF/BMI composites was performed in detail. The pyrolysis and fire parameters were gained and retreated. The following conclusions were drawn:

1. There was only one pyrolysis step of GF/BMI composites in the nitrogen atmosphere, and only the BMI matrix underwent pyrolytic reaction with decomposition gas products consisting mainly of CO_2_, H_2_O, CH_4_, NO_x_, SO_2_, etc. With the increase of heating rate, the initial decomposition temperature, the final decomposition temperature, and the maximum mass loss rate temperature all moved toward high temperature in different degrees. In addition, the average activation energy calculated by the KAS method was up to 156 kJ/mol. The initial decomposition temperature and activation energy were both higher than those of GF/epoxy composites, indicating that GF/BMI composites had good thermal stability.
2. The results of the cone calorimeter test showed that the greater the heat flux, the fuller the combustion, the shorter the time threshold values, and the higher the heat release rate, the total heat release under 25 kW/m^2^ was only 54% of the value under 50 kW/m^2^. Although the LOI gradually decreased with the experimental temperature from 20 to 220 °C, the value always remained no less than 39.0%, reaching the B1 fire resistance level. The smoke density test revealed that GF/BMI composites were superior to GF/epoxy composites in terms of smoke production characteristics. The maximum specific optical density in 4 min was greater, whereas the time to maximum specific optical density and time to critical specific optical density were both shorter in flaming mode than those in non-flaming mode, demonstrating that the flaming mode posed a more serious smoke threat in the early stage of the fire than the non-flaming mode. In general, high fire intensity weakened some retardant properties.
3. The fire performance index, total heat release index, total smoke production index, and toxic gas production index used for evaluating fire hazard showed an overall more serious state with the increase in heat flux. Similarly, the average accumulation rate and the smoke obscuration index were also higher in the flaming mode. The effects of heat and smoke in a fire scene were more intense with the coupling of open flames and thermal radiation.

## Figures and Tables

**Table 1:** The pyrolysis parameters of GF/BMI composites.

β (°C/min)	T_o_ (°C)	T_f_ (°C)	T_p_ (°C)	MLR_p_ (%/min)
5	364	500	424	0.02
10	379	520	441	0.05
20	396	536	457	0.10
30	404	551	470	0.15
40	414	560	481	0.20

**Table 2:** The activation energy of GF/BMI composites available with the KAS method.

α (%)	E (kJ/mol)	R^2^
10	137	0.9896
20	145	0.99471
30	154	0.99754
40	158	0.99596
50	158	0.99883
60	158	0.99794
70	161	0.99957
80	164	0.9986
90	170	0.99685

**Table 3:** Combustion characteristic parameters of GF/BMI composites under different heat fluxes.

Heat Flux (kW/m^2^)	TTI (s)	pHRR (kW/m^2^)	Time-to-pHRR (s)	THR (MJ/m^2^)	TSR (m^2^/m^2^)	RW (%)
25	126	104.9	225	18.6	775.2	79.4
35	85	193.1	157	24.6	844.9	73.8
50	38	242.0	116	34.3	1259.1	65.6

**Table 4:** Fire hazard assessment indicators of GF/BMI composites under different heat fluxes.

Heat Flux (kW/m^2^)	FPI (m^2^ s/kW)	THRI_6min_ (MJ/m^2^)	TSPI_6min_ (m^2^/s)	T_ox_PI_6min_ (g/s)
25	0.56	1.25	2.82	0.18
35	0.54	1.35	2.87	0.57
50	0.33	1.50	3.04	0.69

**Table 5:** LOI of GF/BMI composites at different experimental temperatures.

Temperature (°C)	LOI (%)
20	47.8
50	46.5
100	43.3
150	40.8
220	39.0

**Table 6:** Smoke characteristic parameters and indices of GF/BMI composites in different fire patterns.

Fire Pattern	D_m_	tD_m_ (min)	^4^D_m_	t_16_ (min)	R (1/min)	SOI (1/min)
Flaming	21.64	7.62	21.36	2.56	9.54	48.39
Non-flaming	30.56	19.23	14.63	4.48	3.73	15.27

## Data Availability

Not applicable.
